# Psychometric properties of an Arabic translation of the Nine Item Avoidant/Restrictive Food Intake Disorder Screen (NIAS) in a community sample of adults

**DOI:** 10.1186/s40337-023-00874-0

**Published:** 2023-08-23

**Authors:** Feten Fekih-Romdhane, Rabih Hallit, Diana Malaeb, Fouad Sakr, Mariam Dabbous, Toni Sawma, Sahar Obeid, Souheil Hallit

**Affiliations:** 1 The Tunisian Center of Early Intervention in Psychosis, Department of Psychiatry “Ibn Omrane”, Razi Hospital, 2010 Manouba, Tunisia; 2 Faculty of Medicine of Tunis, Tunis El Manar University, Tunis, Tunisia; 3 School of Medicine and Medical Sciences, Holy Spirit University of Kaslik, P.O. Box 446, Jounieh, Lebanon; 4 Department of Infectious Disease, Bellevue Medical Center, Mansourieh, Lebanon; 5 Department of Infectious Disease, Notre Dame des Secours University Hospital, Postal Code 3, Byblos, Lebanon; 6 School of Pharmacy, Lebanese International University, Beirut, Lebanon; 7 College of Pharmacy, Gulf Medical University, Ajman, United Arab Emirates; 8 School of Arts and Sciences, Social and Education Sciences Department, Lebanese American University, Jbeil, Lebanon; 9 Psychology Department, College of Humanities, Effat University, 21478 Jeddah, Saudi Arabia; 10 Applied Science Research Center, Applied Science Private University, Amman, Jordan; 11 Research Department, Psychiatric Hospital of the Cross, Jal Eddib, Lebanon

**Keywords:** ARFID, NIAS, Picky eating, Fear, Appetite, Psychometric properties, Arabic

## Abstract

**Background:**

No epidemiological data is yet available on Avoidant/Restrictive Food Intake Disorder (ARFID) in Arab countries, which may in part be due to the lack of measures available in Arabic language. This constitutes a major obstacle to further progress of our understanding of the nature, aetiology, course, treatment, and prevention of ARFID, especially as some evidence suggested that ARFID may vary across cultures and food environments. We aimed to contribute to the literature in the eating disorders field by examining the psychometric properties of an Arabic translation of the Nine Item ARFID Screen (NIAS).

**Method:**

This was a cross-sectional, web-based study. A total of 515 Lebanese community adults (mean age of 27.55 ± 10.92 years, 69.9% females) participated. The forward–backward method was adopted to translate the NIAS from English to Arabic.

**Results:**

Confirmatory Factor Analyses provided evidence for the adequate fit indices for the three-factor model (i.e., Picky eating, Fear, and Appetite) and the 9-item version of the NIAS. An adequate reliability of the Arabic NIAS was achieved, with McDonald’s ω ranging from .75 to .90 for the total score and all three subscores. Multi-group analyses demonstrated measurement invariance by sex (males vs. females) and weight groups (underweight/healthy weight [BMI ≤ 25] vs. overweight/obese [BMI > 25]) at the configural, metric, and scalar levels. Adequate patterns of correlations between the NIAS and measures of disordered eating symptoms, psychological distress and well-being were seen. In particular, fear was significantly associated with non-ARFID disordered eating symptoms. Appetite and Picky eating, but not Fear, were inversely correlated with well-being. All three NIAS subscores and the total score were positively correlated with psychological distress.

**Conclusion:**

Findings provided evidence that the Arabic NIAS is a short, valid and reliable self-report measure to screen for ARFID symptoms. In light of these findings, we recommend its use for clinical and research purposes among Arabic-speaking adults.

## Abstract (plain language summary)

Avoidant/restrictive food intake disorder (ARFID) is an eating disorder characterized by food avoidance or dietary restriction associated with at least one of four consequences––weight loss, nutritional deficiency, nutritional supplement dependence, and/or psychosocial impairment. The Nine Item ARFID Screen (NIAS) was developed as a screening measure to detect ARFID symptoms and has nine items with three subscales that map onto symptoms of each ARFID presentation. The NIAS have been translated into multiple languages but not yet validated in Arabic. To that end, we aimed to translate and validate the Arabic version of the NIAS in the present study, which would in turn facilitate improved research and clinical practices related to ARFID in Arabic-speaking nations. The present findings provide support for the psychometric properties of the Arabic version of the NIAS in examining ARFID in Arabic-speaking adults in Lebanon.

## Background

Avoidant/Restrictive Food Intake Disorder (ARFID) is a debilitating eating/feeding disorder that has a broad range of presentations and can be diagnosed at any age. It has been added as a new diagnostic category to the DSM-5 [1] to identify individuals with eating problems that are not driven by distorted body image or fear of weight gain and that can cause significant impairment in psychosocial functioning. Three patterns of eating behaviours that can lead to ARFID symptoms are described: (a) avoidance of foods (e.g., fruits, vegetables, meats) based on their sensory properties (“picky” or selective/neophobic eating); (b) limited interest in eating and/or low appetite; and (c) fear of aversive consequences (e.g., vomiting, choking) from eating [2]. Accordingly, individuals diagnosed with ARFID often fail to meet appropriate energy and/or nutritional needs without the support of nutritional supplements or enteral feeding, thus exhibiting weight loss and substantial nutritional deficiencies [2]. Without an intervention, ARFID may run a chronic course [3], place a significant burden on the patient and their families, and evolve into various medical complications including bradycardia, electrolyte abnormalities, prolonged QT interval, amenorrhoea, lower bone mineral density [4, 5], scurvy [6], loss of vision [7]. Besides, ARFID cases showed high rates of current and lifetime psychiatric comorbidities, including conduct disorders, anxiety and depressive disorders, as well as bipolar-related disorders [8–10].

As ARFID has only recently been introduced to psychiatric nosology, very little is known about the nature of its clinical phenotypic heterogeneity [2]. This has in turn led to limited knowledge about its prevalence estimates [11], as well as the optimal approaches to diagnosis and treatment [12]. A recent systematic review [13] that included 30 studies (including 23 from Western countries) showed that prevalence estimates of ARFID in children and adolescents varied widely, ranging from 0.3 to 15.5% in non-clinical samples, 5–22.5% in clinical samples from specialised paediatric eating disorders treatment settings, and 32–64% in clinical samples from specialised tertiary care services for feeding problems. It is of note, however, that research on the epidemiology of ARFID in adult populations has been until recently “non-existent or highly inconsistent” [14]. Population‐based studies reported prevalence estimates of 0.3–4.8% in general adult populations in Western countries and South‐East Asia [15–18], and of 6.3–11% in clinical adult populations [19, 20]. The effective gathering of prevalence data has long been hindered by the lack of consistent self-report screening and assessment measures to assess ARFID eating patterns in different world languages. Having such measurement instruments is crucial for a timely detection of individuals at-risk for ARFID in clinical contexts, and for enabling to gain a clearer insight into the epidemiology of ARFID in different countries and populations.

Throughout the last few years, some research efforts have been directed at designing and validating measurement instruments that capture ARFID behavioural symptoms. A systematic review published in 2020 [12] could identify a total of four measures that showed promising psychometric properties: (1) the Eating Disturbances in Youth Questionnaire (EDY-Q) [21], (2) the Pica, ARFID and Rumination Disorder Interview (PARDI) [22] and a most updated version the PARDI ARFID Questionnaire [PARDI-AR-Q]) [23], (3) the Eating Disorder Examination (ChEDE) [24], and (4) the Nine Item Avoidant/Restrictive Food Intake Disorder Screen (NIAS) [14]. Among these measures, the NIAS is the briefest self-report measure that has been exclusively designed and validated to explicitly detect the DSM-5-based presentations of ARFID (i.e., sensory sensitivity, fear of aversive consequences, lack of interest) in a community-based adult population [14]. The NIAS demonstrated strong psychometric qualities in terms of divergent, convergent, and discriminant validity in English-speaking adults in the United States [14], in addition to valid cut-off scores to screen for ARFID with good specificity and sensitivity [25]. The NIAS has recently been selected as a recommended measure for ARFID by the International Consortium for Health Outcomes Measurement (ICHOM) [26]. Since its development in 2018, the NIAS has been translated and validated in a few other languages (including Chinese [27] and Spanish [28]) and in different populations (including transgender and non-binary youth and young adults aged 12–23 years [29]). To the best of our knowledge, no Arabic version of the NIAS exists to date. In addition, no epidemiological data is yet available on ARFID in Arab countries. This may constitute a major obstacle to further progress of our understanding of the nature, aetiology, course, treatment, and prevention of ARFID, especially as some evidence suggested that ARFID may vary across cultures and food environments [27, 30]. Therefore, existing data that mostly came from western backgrounds cannot be assumed to apply to other cultural contexts. For instance, some studies showed that ARFID in patients from Eastern backgrounds showed interesting differences in the prevalence and clinical presentation from that in samples from the West (e.g. [20, 31, 32]), which are possibly due to cross-cultural determinants.

To date, there is relatively limited research on disordered eating in Arab populations. A recent literature review on the topic indicated that about one-third of Arab females had restrained eating behaviour [33]. Some studies reported a highly unexpected prevalence of orthorexia nervosa in samples of non-clinical Arab adults [34, 35]. Additionally, cross-cultural research has shown that Arab teenagers were significantly more likely to report anorexia nervosa compared to those from Western cultures [36]. The Arabic culture is expected to shape ARFID symptom manifestations as well as the subsequent experienced problems and impairment for several reasons. The vast majority of Arab people are of Muslim faith, whereas dietary restriction and starvation that may potentially harm the human body are prohibited in Islam (e.g., “Do not throw yourselves with your own hands into destruction”; Surah Al-Baqarah 2: 195). In addition, Arab countries’ traditional social norms and values endorse overeating, as food is closely linked to social/religious events and celebrations. A main characteristic of Arab culture, i.e. hospitality toward a guest, is enacted through food-sharing rituals [37], with interactional moves of insisting and refusing being more conventionalized and more likely to occur between the host and guest in Arab cultures than in Western cultures [38]. Food is treated by Arab people “with the highest respect as it is a sacred blessing with an entrenched social, religious, and cultural meaning” [39]. As such, having ARFID-related eating restrictions in Arab culture and environment, where food plays a central role in people lives, is likely to be problematic and impairing. This underscores the strong need to provide valid and reliable measures that assess ARFID in Arabic-speaking populations. To advance our understanding of ARFID, researchers have also called for more attention to be afforded to adult populations [2] and the subclinical level [27].

For all these reasons, we sought through this study to examine the psychometric properties of an Arabic translation of the NIAS. We hypothesized that the Arabic NIAS will: (1) replicate the originally proposed three-factor structure, (2) show good composite reliability and measurement invariance by sex (males vs. females) and weight groups (underweight/healthy weight [BMI ≤ 25] vs. overweight/obese [BMI > 25]), and (3) demonstrate adequate patterns of correlations between the NIAS and measures of disordered eating symptoms, psychological distress and well-being. In particular, we expected that all three NIAS scales will be positively correlated with disordered eating symptoms. Indeed, although symptoms related to ARFID are theorized to be distinct from “traditional” disordered eating symptoms related to anorexia nervosa and bulimia nervosa [1], research has suggested a potential overlap between these two conditions [40]. ARFID symptoms have been proposed to “share endophenotypes with symptoms of other eating disorders, simultaneously increasing risk for all forms of disordered eating”, and/or “precipitate additional eating pathology” [40]. A previous psychometric study [25] demonstrated that the NIAS is an effective tool in distinguishing between individuals with ARFID and those with other eating disorders symptoms, albeit with a high overlap between the two groups on the NIAS-fear and NIAS-appetite subscales. Authors suggested that ARFID and other eating disorders appear to share transdiagnostic features [25]. Furthermore, we expected that ARFID symptoms will be positively associated with psychological distress and negatively associated with well-being. We also predicted to replicate Zickgraf and Ellis’ findings that only Appetite will be negatively related to BMI.

## Methods

### Translation and adaptation procedures

Before their use in the current study, the ARFID scale was translated and adapted to the Arabic language and context. To this end, it was translated to the literary Arabic language (i.e., Modern Standard Arabic), which represents the official language of all Arab countries, and is used to communicate between speakers of different groups. The purpose of achieving semantic equivalence between measures in their original and Arabic versions following international norms and recommendations [41]. For this, the forward and backward translation method was applied. The English version was translated to Arabic by a Lebanese translator who was completely unrelated to the study. Afterwards, a Lebanese psychologist with a full working proficiency in English, translated the Arabic version back to English. The translation team ensured that any specific and/or literal translation was balanced. The initial and translated English versions were compared to detect/eliminate any inconsistencies and guarantee the accuracy of the translation by a committee of experts composed of two psychiatrists and one psychologist, in addition to the research team and the two translators [42]. An adaptation of the measure to our specific context was performed, and sought to determine any misunderstanding of the items wording as well as the ease of items interpretation; and, therefore, ensure the conceptual equivalence of the original and Arabic scales in both contexts [43]. After the translation and adaptation of the scale, a pilot study was done on 30 patients to ensure all questions were well understood; no changes were applied after the pilot study.

### Measures

#### Avoidant/Restrictive Food Intake Disorder screen (NIAS)

This scale was designed to screen for ARFID. It is composed of 9 items, scored on a 6-point Likert scale, “Strongly disagree,” “Disagree,” “Slightly disagree,” “Slightly agree,” “Agree,” and “Strongly agree” [e.g. *I am a picky eater* (item1); *I dislike most foods that other people eat easily* (item 2)] [14]. It yields three subscales composed of 3 items each as follows: Picky eating, Appetite and Fear. Higher scores indicate more avoidant/restrictive eating. Cutoff values of ≥ 10, ≥ 9, and/or ≥ 10 have been proposed for capturing individuals who fit the NIAS dimensions Picky eating, Appetite, and Fear, respectively [25].

#### Eating Attitude Test (EAT-7)

This scale is the Arabic shortened version of the Eating Attitude Test-26 (EAT-26) [44, 45]. It is composed of 7 items scored on a 6-point Likert scale [46] [e.g*. Aware of the energy content of foods that I eat* (item2); *Avoid foods with sugar in them* (item 5)]. Higher scores reflect more severe disordered eating symptoms related to anorexia nervosa and bulimia nervosa (ω = 0.84).

#### Depression anxiety and stress scale-8 items (DASS-8)

Validated in Arabic [47], this scale is composed of 8 items that measure depression (3 items), anxiety (3 items) and stress (2 items) [e.g. *I felt that I was using a lot of nervous energy* (item 1); *I felt down-hearted and blue* (item 5)]. Questions are rated on a 4-point Likert scale (“0 = does not apply to me to “3 = always applies to me”). Higher scores reflect more psychological distress (ω = 0.89).

#### WHO-wellbeing scale

Validated in Arabic [48, 49], this scale is composed of 5 items [e.g. *I have felt cheerful in good spirit, in the last 2 weeks* (item 1)], scored on a 6-point Likert scale (“0 = at none time to 5 = all of time”), with higher scores reflecting better wellbeing [50] (ω = 0.93).

#### Demographics

Participants were asked to provide their demographic details consisting of age, sex, and education level. Weight and height were self-reported by participants to calculate the Body Mass Index (BMI); the latter was later subdivided into underweight/normal (Body Mass Index [BMI] ≤ 25) and overweight/obese (BMI > 25) [51].

### Procedures

All data were collected via a Google Forms link; the sample was recruited conveniently between February and March 2023. The survey link was sent using social media applications (WhatsApp, Instagram, Messenger) and included an estimated duration. Inclusion criteria for participation included: (1) being of a resident and citizen of Lebanon, (2) aged 18 years and above, (3) having access to the Internet, and (4) willing to participate in the study. Excluded were those who refused to fill out the questionnaire. Internet protocol (IP) addresses were examined to ensure that no participant took the survey more than once. After providing digital informed consent, participants were asked to complete the instruments described above, which were presented in a pre-randomised order to control for order effects. The survey was anonymous and participants completed the survey voluntarily and without remuneration, in approximately 20 min on average [52].

### Analytic strategy

#### Confirmatory factor analysis (CFA)

There were no missing responses in the dataset. We used data from the total sample to conduct a CFA using the SPSS AMOS v.29 software. As a rule of thumb, simulation studies show that with normally distributed indicator variables and no missing data, a reasonable sample size for a simple confirmatory factor analysis model is about N = 150 [53], which was exceeded in our sample. Our intention was to test the original model of the ARFID scale (i.e., three-factor model). Parameter estimates were obtained using the maximum likelihood method and fit indices. For this purpose, the normed model chi-square (χ^2^/*df*), the Steiger-Lind root mean square error of approximation (RMSEA), the Tucker-Lewis Index (TLI) and the comparative fit index (CFI). Values ≤ 5 for χ^2^/*df*, and ≤ 0.05 for RMSEA, and 0.95 for CFI and TLI indicate good fit of the model to the data [54]. Additionally, evidence of convergent validity was assessed in this subsample using the Fornell-Larcker criterion, with average variance extracted (AVE) values of ≥ 0.50 considered adequate [55]. The absence of multicollinearity was verified through tolerance values > 0.2 and variance inflation factor (VIF) values < 5. Multivariate normality was not verified at first (Bollen-Stine bootstrap *p* = 0.002); therefore, we performed non-parametric bootstrapping procedure (available in AMOS).

#### Sex and weight invariance

To examine sex and weight invariance of ARFID scores, we conducted multi-group CFA [56] using the total sample. Measurement invariance was assessed at the configural, metric, and scalar levels [57]. Following the recommendations of Cheung and Rensvold [58] and Chen [56], we accepted ΔCFI ≤ 0.010 and ΔRMSEA ≤ 0.015 or ΔSRMR ≤ 0.010 (0.030 for factorial invariance) as evidence of invariance. The Student t test was used to compare two means in case of evidence of measurement invariance.

#### Further analyses

Composite reliability in both subsamples was assessed using McDonald’s ω, with values greater than 0.70 reflecting adequate composite reliability [59]. McDonald’s ω was selected as a measure of composite reliability because of known problems with the use of Cronbach’s α (e.g., [60]). The social support total score was considered normally distributed since the skewness (= 0.406) and kurtosis (= − 0.270) values varied between ± 1 [61]. We examined bivariate correlations between the ARFID and the DASS-8 and WHO-5 using the Pearson test. Based on Cohen [62], values ≤ 0.10 were considered weak, ~ 0.30 were considered moderate, and ~ 0.50 were considered strong correlations.

## Results

### Participants

Five hundred fifteen participants participated in this study, with a mean age of 27.55 ± 10.92 years, 69.9% females and 83.7% with a university level of education. Moreover, the mean BMI was 24.27 ± 4.54 kg/m^2^; 189 (36.7%) were overweight/obese. Moreover, 56 (10.9%) had NIAS-picky eating scores ≥ 10, 112 (21.7%) NIAS-appetite scores ≥ 9 and 49 (9.5%) NIAS-fear scores ≥ 10. Finally, 17 (3.3%) had positive screen on any NIAS subscale (≥ 10 NIAS-picky eating, ≥ 9 NIAS-appetite, and ≥ 10 NIAS-fear).

### Confirmatory factor analysis of the ARFID scale

CFA indicated that fit of the three-factor model of the ARFID scale was acceptable: χ^2^ = 75.55, *df* = 24 (*p* < 0.001), RMSEA = 0.065 (90% CI 0.049, 0.081), SRMR = 0.036, CFI = 0.978, TLI = 0.967. The standardised estimates of factor loadings were all adequate (see Table 1). The convergent validity for this model was good, as AVE = 0.61.

**Table 1:** Standardized factor loadings derived from the confirmatory factor analysis (CFA) of the avoidant/restrictive food intake disorder in the total sample

Item	Loading factor
*Factor 1: Picky eating*	
1. I am a picky eater	.60
2. I dislike most foods that other people eat easily	.78
3. The list of foods that I will eat is shorter than the list of foods I won’t eat	.72
*Factor 2: Appetite*	
4. I am not very interested in eating; I seem to have a smaller appetite than other people	.78
5. I have to push myself to eat regular meals throughout the day, or to eat a large enough amount of food at meals	.68
6. Even when I am eating a food I really like, it is hard for me to eat a large enough volume at meals	.80
*Factor 3: Fear*	
7. I avoid or put off eating because I am afraid of discomfort, choking, or vomiting	.85
8. I restrict myself to certain foods because I am afraid that other foods will cause discomfort, choking, or vomiting	.86
9. I eat small portions and/or infrequent meals because I am afraid of discomfort, choking, or vomiting	.89

### Measurement invariance

As reported in Table 2, all indices suggested that configural, metric, and scalar invariance was supported across sex and weight. The results showed that there was no statistically significant difference between males and females in all ARFID dimensions. Furthermore, no significant difference was found between participants with underweight/healthy weight vs overweight/obese except for the appetite subscale score where non-overweight participants scored higher than those who are overweight/obese (Table 3).

**Table 2:** Measurement invariance of the ARFID across sex and body mass index in the total sample

Model	χ^2^	*Df*	CFI	RMSEA	SRMR	Model comparison	Δχ^2^	ΔCFI	ΔRMSEA	ΔSRMR	Δdf	*p*
*Model 1: Invariance by sex*												
Configural	107.62	48	.974	.049	.051							
Metric	113.34	54	.974	.046	.057	Configural versus metric	5.72	< .001	.003	.006	6	.455
Scalar	118.16	61	.975	.043	.059	Metric versus scalar	4.82	.001	.003	.002	7	.682
*Model 2: Invariance by body mass index*												
Configural	132.27	48	.964	.059	.042							
Metric	142.41	54	.962	.056	.041	Configural versus metric	10.14	.002	.003	.001	6	.118
Scalar	145.26	61	.964	.052	.042	Metric versus scalar	2.85	.002	.004	.001	7	.898

*CFI* comparative fit index, *RMSEA* Steiger-Lind root mean square error of approximation, *SRMR* standardised root mean square residual

**Table 3:** Comparison between sex and weight groups in terms of the ARFID total scale and subscales scores in the total sample

	ARFID total score	Picky eating	Appetite	Fear
*Sex*				
Males	16.17 ± 8.68	5.94 ± 3.18	5.60 ± 3.37	4.64 ± 3.47
Females	15.41 ± 8.40	5.64 ± 3.17	5.26 ± 3.41	4.51 ± 3.54
*T*	.943	.964	1.046	.394
*Df*	513	513	513	513
*P*	.346	.336	.296	.694
Effect size	.089	.094	.100	.037
*Weight*				
Underweight/healthy weight	16.15 ± 8.38	5.87 ± 3.18	5.61 ± 3.45	4.67 ± 3.52
Overweight/obese	14.75 ± 8.60	5.49 ± 3.15	4.93 ± 3.23	4.34 ± 3.51
*T*	1.808	1.326	2.219	1.018
*Df*	513	513	513	513
*P*	.071	.186	**.027**	.309
Effect size	.164	.120	.203	.094

Numbers in bold indicate significant *p* values

### Composite reliability

Composite reliability of scores was adequate in the total sample for the ARFID total scale (ω = 0.88), picky eating (ω = 0.75), appetite (ω = 0.80) and fear (ω = 0.90) subscales.

### Associations of NIAS with other measures

Higher NIAS scores and sub-scores were significantly correlated with higher psychological distress, lower wellbeing. The NIAS fear subscale was significantly associated with more disordered eating as measured by the EAT-7 (Table 4).

**Table 4:** Correlations of the NIAS total scores and sub-scores with the other measures in the total sample

	Mean ± SD	1	2	3	4	5	6	7	8
1. NIAS total score	15.64 ± 8.48	1							
2. NIAS: picky eating	5.73 ± 3.17	.79***	1						
3. NIAS: appetite	5.36 ± 3.40	.87***	.52***	1					
4. NIAS: fear	4.55 ± 3.51	.86***	.49***	.66***	1				
5. EAT-7	3.69 ± 4.42	.06	− .01	.04	.11*	1			
6. DASS-8	11.16 ± 6.72	.25***	.22***	.19***	.21***	.02	1		
7. Wellbeing	14.08 ± 5.60	− .10*	− .12**	− .09*	− .05	.14**	− .38***	1	

*EAT-7* eating attitudes test 7 items, *DASS-8* depression, anxiety and stress scale 8 items

*p < .05; **p < .01; ***p < .001; values reflect Pearson correlation coefficients

## Discussion

No data exists to date on ARFID in Arab countries, which may in part be due to the lack of measures available in Arabic language. We aimed to contribute to the literature in the eating disorders field by validating the Arabic version of the NIAS in a sample of non-clinical Arabic-speaking adults (N = 515) from Lebanon. As expected, analyses revealed that the Arabic NIAS yielded a three-factor solution, which showed excellent levels of reliability. In addition, the Arabic NIAS demonstrated measurement invariance across gender and BMI, as well as good correlations with the other measures. In light of these findings, we recommend its use among Arabic-speaking adults. Offering this psychometrically sound Arabic version of the NIAS may help to provide accurate epidemiological data on ARFID in Arab countries, increase the awareness of ARFID screening and diagnosis in Arab settings, and inform the development of culturally-tailored, evidence‐informed interventions.

CFA provided evidence for the adequate fit indices for the three-factor model (i.e., Picky eating, Fear, and Appetite) and the 9-item version of the NIAS, which further supports the DSM-5-oriented ARFID subdomains proposed in the original scale [2]. Other linguistic validations of the NIAS, including the Chinese version [27] in college students and the Spanish version [28] in a Mexican adolescent and young adult population. More recently, Zickgraf et al. [29] could also replicate the original factorial structure in English-speaking sexual minority youth. Furthermore, an adequate reliability of the Arabic NIAS was achieved, with McDonald’s ω ranging from 0.75 to 0.90 for the total score and all three subscores. Similarly, a good reliability of the NIAS was demonstrated in the original [14] and subsequent validations (e.g., Cronbach’s α of 0.73–0.86 in Chinese college students [27], McDonald’s ω of 0.70–0.90 in Mexican youth [28]).

Another finding of our study is that the factor loadings of the Arabic NIAS remained invariant by gender and weight groups at the three levels (configural, metric, and scalar). Evidence for invariance reflects that across-group comparisons of NIAS subscale means is valid. In other words, individuals of both genders and different weight groups understand and interpret the meaning of NIAS items in the same way. In line with our findings, Medina‐Tepal et al. [28] provided evidence supporting measurement invariance across sex. Between-sex comparisons revealed no statistically significant differences in all three ARFID presentations in our sample. The previous literature on sex difference in ARFID manifestations has yielded conflicting results. In Chinese college students, no significant sex differences were found except for the subscale “Appetite”, with males scoring significantly higher than females [27]. In American transgender and non-binary youth, assigned females at birth displayed greater scores in Fear and Appetite subscales than assigned males at birth [29]. Previous data in children and adolescent samples also showed mixed results, with rates of ARFID being higher either in males [63–65] or females [66–68]. As for comparisons across weight groups, findings showed no significant differences between individuals belonging to underweight/healthy weight and overweight/obese groups, except for Appetite scores which were lower in the latter group. These findings were expected, and were consistent with those of the original validation study [14], thus suggesting that the NIAS captures eating behavioural patterns related to significant and/or prolonged inadequate intake which ultimately results in weight loss or lack of weight gain. Different findings were reported by He et al. [27], who found that underweight Chinese students exhibited significantly higher scores in Appetite, Picky eating and NIAS total scores. These differences were explained by cultural factors, as authors suggested that adult picky eating might have protective effect against overweight/obesity in China, a cultural context where there is lower prevalence of and fewer environmental contributors to overweight/obesity than Western contexts (e.g., the United states [US]) [27]. All these controversial data indicate the need for more investigations of ARFID characteristics across sex and weight groups in different settings and countries.

Similar to the original validation [14], we used the EAT as a measure of non-ARFID disordered eating to explore divergent validity. Findings indicated that only Fear was significantly associated with disordered eating symptoms. It is of note that there is scant research to date on how disordered eating (such as anorexia nervosa, bulimia nervosa and binge eating) relates to ARFID symptoms. Some evidence suggests that picky eaters are at an increased risk of developing anorexia nervosa [69], although a lack of association between the two entities has also been observed [70]. In the original validation study, the NIAS Fear and Picky eating (but not Appetite) subscores were independently correlated with EAT-26 scores [14]. A positive relationship between picky eating and disordered eating symptoms has also been previously observed among adults (e.g. [71, 72]). Burton Murray et al. [25] also found a significant overlap between ARFID and traditional eating disorders symptoms related to anorexia nervosa and bulimia nervosa, as these two disorders involve shared manifestations. Indeed, the ARFID Fear of aversive consequences may share overlapping presentations with other disordered eating symptoms, as both often include gastrointestinal symptoms [19, 73].

Our findings also revealed that Appetite and Picky eating, but not Fear were inversely correlated with well-being. In addition, all three NIAS subscores and the total score were positively correlated with psychological distress. Consistently, Zickgraf and Ellis [14] found a positive association between Fear and anxiety as well as between Appetite and depression in the US validation sample. He et al. [27] showed that Fear and Appetite were each independently associated with psychological distress in the Chinese validation sample. The lack of significant correlation between Fear and well-being in our sample may be partly explained by cultural factors. The way how individuals perceive aversive consequences of eating (such as vomiting or choking) may be influenced by cultural factors, and can therefore differently affect well-being across cultural backgrounds. In collectivistic cultures, such as Arab countries, somatic symptoms seem to reflect a “constructive response” to distress; where, generally, individuals “explicitly exhibit somatization in response to life stressors” [74]. Indeed, the prevalence of medically unexplained somatic symptoms is consistently reported to be higher in Arab people than globally, with gastrointestinal symptoms being among the most commonly reported [75].

### Study limitations and research perspectives

Certain limitations should be considered when interpreting the findings and conclusions of this study. Our data were gathered using a convenience (non-probabilistic) and web-based sampling methods, which may limit the generalization of the present findings. We did not implement practices to ensure the integrity of the data (embedding attention checks throughout the survey) and did not have the option to check the response time of participants with Google forms. In addition, we used a self-report survey, meaning the answers could be affected by recall or social desirability biases. Another limitation is that our sample was disproportionate in terms of sex and educational levels (with the majority of participants being females of a high educational level), which may have impacted the findings. Also, our study relied on an adult non-clinical sample to validate the NIAS. Additional studies are required to test its psychometric properties in clinical samples (e.g., patients with eating disorders) and across the lifespan. As cross-national cultural differences may also exist between Arab countries, one should be cautious in generalizing the findings to the broader Arabic-speaking community in other parts of the world. To address this limitation, future validations in contexts other than Lebanon are needed to further confirm the robustness of the scale across various Arab backgrounds. Other important psychometric characteristics of the NIAS (e.g., test–retest reliability) have not been explored in the present study, and still need to be considered in future studies. Finally, further research still needs to identify clinical cut-offs on the Arabic NIAS, in order to enable to define individuals at-risk for ARFID and determine prevalence rates of ARFID in Arab populations.

## Conclusion

The present study is the first to investigate ARFID symptoms in an Arab population from a developing country of the Middle East and North Africa (MENA) region. Findings provided evidence that the Arabic NIAS is a short, valid and reliable self-report measure to screen for ARFID among Arabic-speaking adults from the general population. Pending larger scale studies in other settings, countries and age groups, we recommend its use to screen for ARFID symptoms, at least among Arabic-speaking adults in non-clinical settings and the Lebanese context. We hope that by providing a psychometrically sound Arabic version of the NIAS we can aid in fostering cross-cultural research on ARFID by including under-represented populations from non-Western non-developed countries, and furthering the understanding of this new diagnostic entity.

## Data Availability

All data generated or analyzed during this study are not publicly available due the restrictions from the ethics committee, but are available upon a reasonable request from the corresponding author (SH).
